# Massachusetts

**Published:** 1896-04

**Authors:** Howard P. Rogers

**Affiliations:** Secretary


### MASSACHUSETTS VETERINARY ASSOCIATION.

THE monthly meeting of the Association was held at 19 Boylston Place, Boston, January 21, 1896, with a good attendance. Drs. Langdon Frothingham, of Boston, and A. Cannon, of Malden, were elected members. The application of Dr. J. Marshall was received. Dr. Parker read a paper on "Milk as a Factor in the Causation of Disease."[1] A general discussion followed, in which Drs. Cooper Curtice, Osgood, Burr, and McLaughlin took part.

HOWARD P. ROGERS,
Secretary.

[1] Printed in this number.

